# Crystal structure of the CupB6 adhesive tip from the chaperone-usher family of pili from *Pseudomonas aeruginosa*

**DOI:** 10.1016/j.bbapap.2016.07.010

**Published:** 2016-11

**Authors:** Masooma Rasheed, James Garnett, Inmaculada Pérez-Dorado, Daniela Muhl, Alain Filloux, Steve Matthews

**Affiliations:** a Department of Life Sciences, Imperial College London, London SW7 2AZ, United Kingdom; b Queen Mary University of London, Department of Chemistry and Biochemistry, School of Biological and Chemical Sciences, Joseph Priestley Building, Mile End Road, London E1 4NS, United Kingdom

**Keywords:** CupB6, Chaperone-usher, Adhesin, From *Pseudomonas aeruginosa*

## Abstract

*Pseudomonas aeruginosa* is a Gram-negative opportunistic bacterial pathogen that can cause chronic infection of the lungs of cystic fibrosis patients. Chaperone-usher systems in *P. aeruginosa* are known to translocate and assemble adhesive pili on the bacterial surface and contribute to biofilm formation within the host. Here, we report the crystal structure of the tip adhesion subunit CupB6 from the *cupB1–6* gene cluster. The tip domain is connected to the pilus via the N-terminal donor strand from the main pilus subunit CupB1. Although the CupB6 adhesion domain bears structural features similar to other CU adhesins it displays an unusual polyproline helix adjacent to a prominent surface pocket, which are likely the site for receptor recognition.

## Highlights

• Crystal structure of the tip adhesion subunit CupB6 from the cupB1-6 gene cluster of *Pseudomonas aeruginosa*
• CupB6 possesses an atypical adhesion domain connected to a canonical chaperone-usher pilus subunit
• CupB6 caps the pilus shaft via donor strand complementation with the N-terminus of CupB1
• CupB6 possesses unusual polyproline helices adjacent to a prominent surface pocket

## 1 Introduction

*Pseudomonas aeruginosa* is a Gram-negative opportunistic bacterial pathogen that can cause chronic infection of the lungs of cystic fibrosis patients. It can persist in tissues in the form of specialized bacterial communities, known as a biofilm, which are formed through interactions between surface component on bacterial cells and to surfaces. *P. aeruginosa* is also often used as a model organism for the study of biofilm formation [1]. By screening a mutant library of *P. aeruginosa* strains, a series of genes were identified to be important in early stages of biofilm formation. Several clusters of these genes showed sequence similarity with the chaperone/usher (CU) pathway [2], [3], which is conserved in Gram negative bacteria and involved in the assembly of extracellular fimbriae. CU systems possess an outer-membrane transporter called the usher, a periplasmic chaperone, and a variety of bespoke fimbrial subunits. Once fimbrial subunits are translocated into the periplasm, they are captured and stabilized by the chaperone before subsequent targeting to the usher for secretion to the bacterial surface. The first fimbrial component to be presented to the pore for secretion is the tip subunit and these often comprise an N-terminal adhesion domain directly coupled to a canonical pilus immunoglobulin (Ig) domain. The pilus domain lacks the final G-strand necessary to complete an Ig fold, however this is provided by the incoming subunit by donating this as a flexible N-terminal extension (NTE). The subsequent interaction of the NTE with the vacant G-strand groove and neighboring β-strands provide the highly stable mode of polymerization [4].

It has been shown that chaperone-usher systems in *P. aeruginosa* [5] play important roles in biofilm formation by presenting adhesive pili on the bacterial surface. One of these clusters, encoded by the *cupB* operon, is unique as it contains a non-chaperone-usher gene product, CupB5, which is related to the TpsA-like substrates of two-partner secretion (TPS) systems [6]. Furthermore, it possesses two cognate chaperones CupB2 and CupB4, which is unusual for CU systems, as the only other system from another bacterial species that is reported with more than one periplasmic chaperone is the *E. coli* common pilus [7]. The recent crystal structure of CupB2 revealed the classical chaperone architecture of two Ig-like domains that interacts with the main pilus subunit CupB1 via donor strand complementation [8]. The CupB4 chaperone was shown to be associated with the tip adhesin CupB6, which is larger than classical adhesive tip subunits and displays only limited sequence identity with other CU subunits. Despite the involvement of the *P. aeruginosa* CU fimbrial structures in biofilm formation via mutual interactions between bacteria [9], there are no published structures of the adhesive elements. In this paper we describe the crystal structure of the tip adhesin CupB6. We show that is possesses an atypical adhesion domain connected to a canonical CU pilus subunit CupB1 via the N-terminal donor strand complementation. While the adhesive domain of CupB6 bears general features akin to other CU adhesins it possesses an unusual polyproline helix adjacent to a prominent surface pocket, which is likely to represent the binding side for its cognate receptor.

## 2 Materials and methods

### 2.1 Cloning and protein expression

Full length CupB6 sequence (39–343) minus the N-terminal signaling sequence (residues 1–38) was amplified by PCR using *P. aeruginosa,* PAO1 gDNA as a template. A reverse primer was used to introduce a SDNK tetrapeptide linker at the C-terminus of CupB6 protein, followed by the N-terminal donor strand (residues 1–13) of the major CupB1 pilin protein. The donor strand complemented product referred to here as CupB6_dscB1_ was cloned into a pET28b vector encoding a C-terminal His_6_ tag. *E. coli* Shuffle T-7 strain (NEB) were transformed with recombinant plasmid harboring the CupB6_dscB1_ gene. Expression was carried out it either in LB media (50 mg/l kanamycin) for unlabeled CupB6, or M9 minimal media (50 mg/l kanamycin) supplemented with 0.4% glucose, 2 μg/ml thiamine, 2 mM MgSO_4_, 100 μg/ml each of Lysine, Phenylalanine and Threonine and 50 μg/ml each of Isoleucine, Leucine, Valine and L-Selenomethionine for Se-labelling CupB6. For either native CupB6 or Se-labelling bacterial cultures were grown at 37 °C and the expression was induced when an OD600 of ~ 0.8 was reached with 0.1 mM isopropyl β-d-1-thiogalactopyranoside (IPTG), followed by overnight incubation at 18 °C. The cultures were harvested at 5000 g for 10 min at 4 °C and cell pellets were stored at − 20 °C for purification.

### 2.2 Protein purification and crystallization

For the production of all CupB6 samples, the cell pellets were re-suspended in 20 mM Tris pH 8.0, 300 mM NaCl, lysed with a cell disrupter and centrifuged at 15,000*g* for 1 h at 4 °C. The supernatant was purified with nickel affinity chromatography and finally gel filtered using a Superdex 200 column (GE Healthcare) pre-equilibrated in 20 mM Tris-HCl pH 8.0, 100 mM NaCl. The eluted CupB6_dscB1_ samples were concentrated up to 17 mg/ml and initial crystallization conditions were screened by sitting-drop vapour diffusion method at 293 K using sparse-matrix crystallization kits (Hampton Research, USA; Emerald BioSciences, USA; Molecular Dimensions Ltd., USA) in MRC 96-well optimization plates (Molecular Dimensions, USA). Droplets consisted of 100 nl protein solution and 100 nl reservoir solution and were set up using a Mosquito nanolitre high-throughput robot (TTP Labtech). CupB6_dscB1_ protein was crystallized in 1.6 M NaH_2_PO_4_/0.4 M KH_2_PO_4_, 100 mM phosphate citrate pH 4.2 after three days.

### 2.3 X-ray data collection and processing

Crystals were briefly washed in reservoir solution supplemented with 25% *v*/*v* glycerol, mounted in cryoloops (Hampton) and then immediately flash cooled in liquid nitrogen. Data were collected for CupB6_dscB1_ crystals on using a PILATUS 6M detector on beamline I02 at the Diamond Light Source (DLS), England. Diffraction data were processed using MOSFLM [10] and scaled with AIMLESS [11], within the Xia 2 package [12]. The content of the unit cell was analyzed using the Matthews coefficient [13]. The phase evaluation and density modification for MAD data were carried out in SHARP. The overall figures of merit for acentric and centric reflections were 0.15964 and 0.16471, respectively. The (correlation on E^2^)/contrast increased from 2.4606 before density modification to 3.3788 after. An initial model was built by using BUCCANEER [14] and refinement was carried out in REFMAC [15] using TLS, secondary structure, and NCS restraints, with model building carried out in COOT [16]. Processing and refinement statistics for the final model can be found in Table 1.

### 2.4 Accession numbers

Coordinates and structure factors for the CupB6_dscB1_ models have been deposited in the Protein Data Bank (PDB code 5CYL).

## 3 Results and discussion

**Fig. 1:**
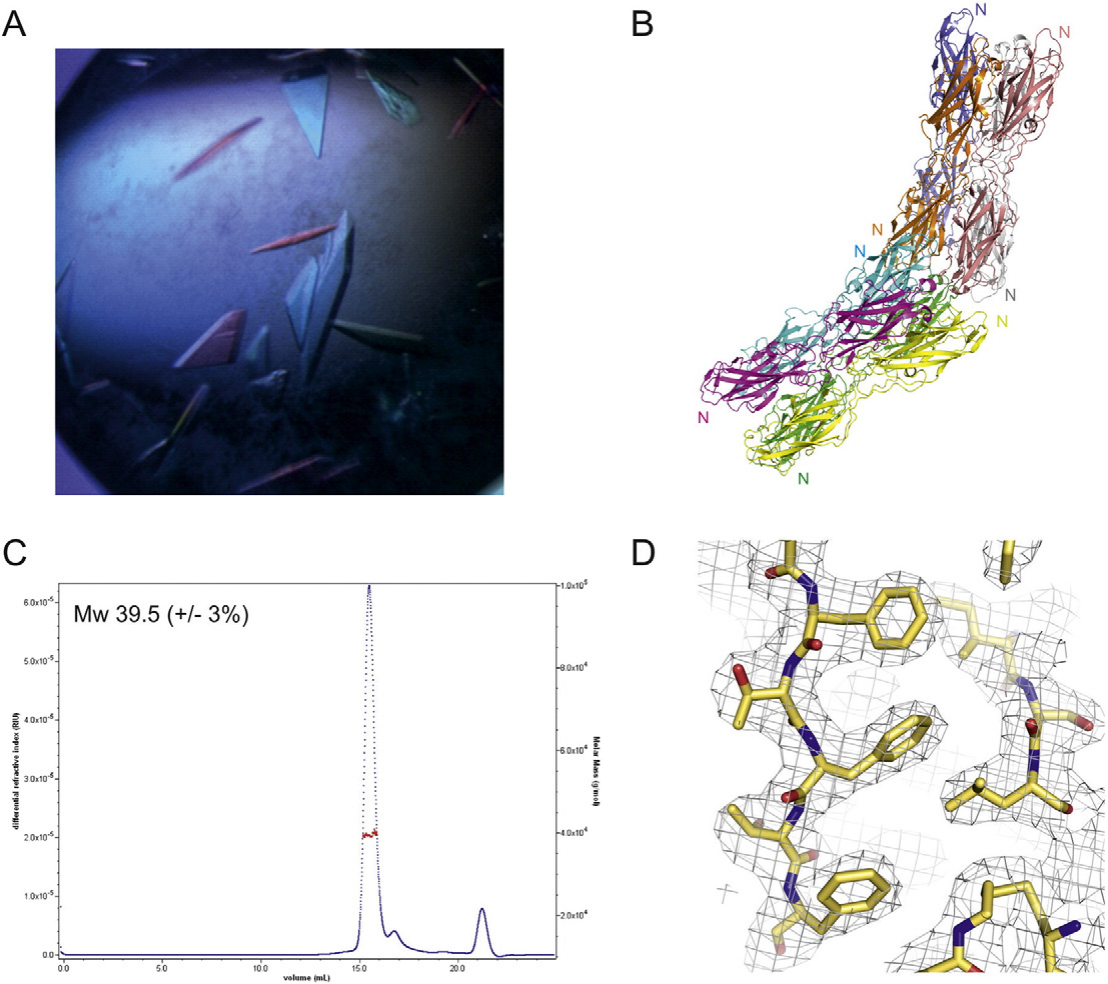
Crystallization and characterization of CupB6_dscB1_. (A) Crystals of CupB6_dscB1_. (B) The asymmetric unit for the crystal structure of CupB6_dscB1_ showing the arrangement of the two up-down-up-down tetramers. The approximate location of the N-termini for each chain is indicated. (C) SEC MALS analysis of CupB6_dscB1_. The results indicate that the protein is monomeric with a deduced molecular mass of ~ 39 kDa. (D) Example of electron density map obtained for CupB6_dscB1_ and the fit of the polypeptide which corresponds to the 2F_o_–F_c_ electron density map after refinement at 2.77 Å, with sigma = 1.0.

CupB6_dscB1_ was crystallized in 1.6 M NaH_2_PO_4_/0.4 M KH_2_PO_4_ 100 mM phosphate citrate pH 4.2 after three days of incubation at 293 K. The structure was determined by Se-MAD and then phase extended to 2.91 Å from native crystals. The crystals belong to *C2* space group and the asymmetric unit consists of eight molecules composed from two up-down-up-down tetramers (Fig. 1A, B). The eight protein molecules are essentially identical with an average RMSD of 0.35 Å. The protein oligomerization state was determined by size exclusion chromatography using a Superdex 200 HR 10/30 column coupled to multiple laser light scattering (SEC-MALLS). The molecular mass of CupB6_dscB1_ in solution was determined to be 39.5 kDa, which is consistent with a monomer (Fig. 1C). The averaging of density for non-crystallographic symmetry related copies of the molecule, reducing noise and increasing the restraints on the phases of electron density maps, significantly improved the overall electron density (Fig. 1D) and allowed us to construct the majority of the residues for all the eight chains. No density was observed for the vector-encoded C-terminal histidine tag; residues 0–2 in chain A; residues 0, 344–348 and 362–363 in chain B; residues 0–3, 16, 49,53, 93, and 361–363 in chain C; residues 0, 323, 324, and 362–363 in chain D; residues 0–3 and 343–347 in chain E; residues 0–2, 196, 210–211, 221, 227, 276–277, 323–325, 334, 343–349 and 362–363 in chain F; residues 331, 335,336, 345–348 and 361–363 in chain G; residues 0, 344–347 in chain H.

**Fig. 2:**
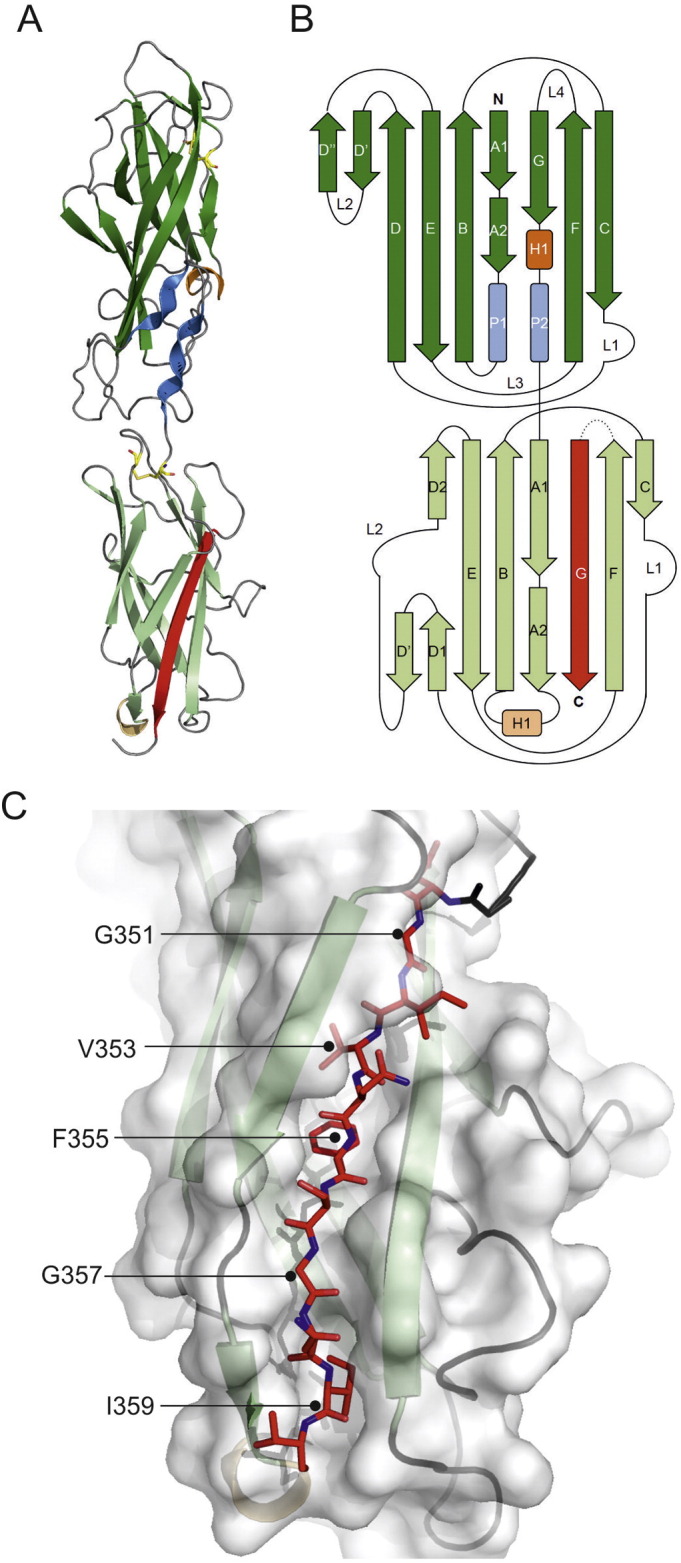
Overall structure of CupB6_dscB1_. (A) Cartoon representation of CupB6 with strands, α-helices and polyproline helices colored in green, orange and blue respectively. The N-terminal adhesin domain is shaded in darker colors than the pilin domain. The self-complementing donor strand from CupB1 is shown as a red arrow. (B) Topology diagram of CupB6_dscB1_ using the same color scheme as in (A). (C) Surface representation of the CupB6 pilin domain with self-complementing donor strand from CupB1 as red sticks. Residues for interacting side-chains in dscCupB1 are indicated.

**Fig. 3:**
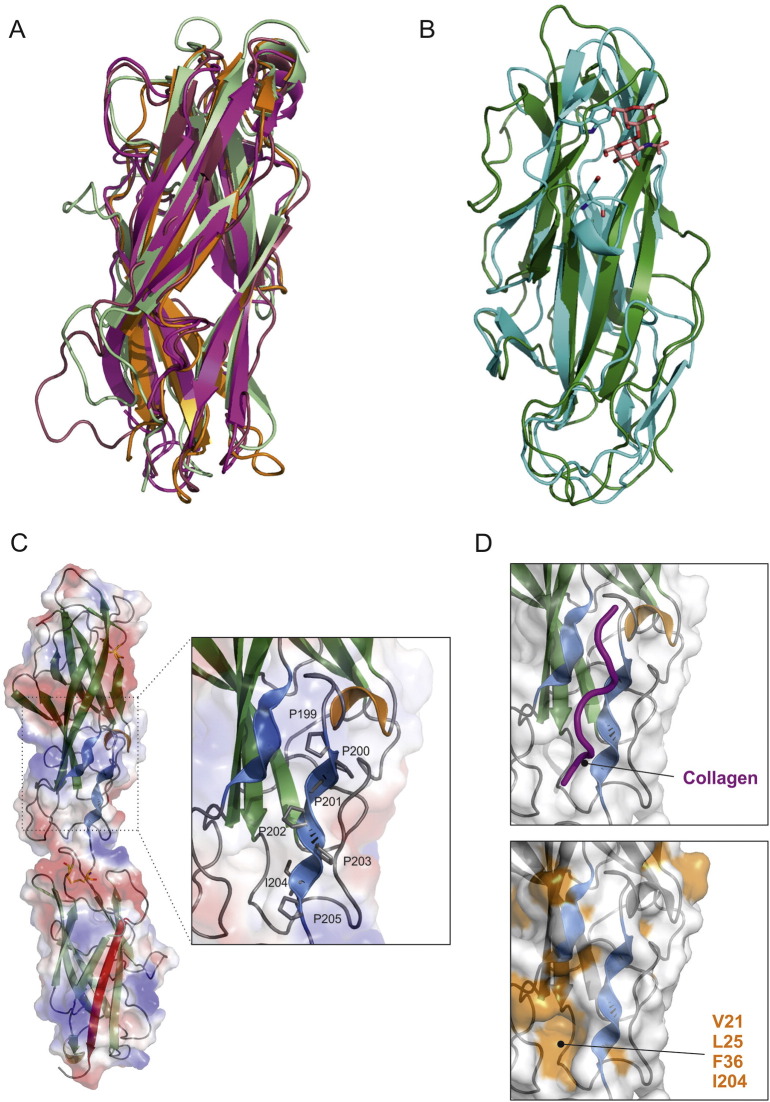
Comparison of CupB6_dscB1_ with other chaperone/usher subunits. (A) Cartoon representation of the CupB6_dscB1_ pilin domain (light green) with FimF (pdb: 2jmr in dark red), FimA (pdb: 2jty in magenta) and FimG (pdb:3bfw in orange) from *E. coli* type I fimbriae. (B) Cartoon representation of the CupB6_dscB1_ adhesin domain (dark green) with F17b-G lectin domain with bound GlcNAc(beta1–3)Gal (pdb: 4k0O in cyan), FimA (pdb: 2jty in magenta). The GlcNAc(beta1–3)Gal ligand and key conserved interacting residues are shown as pink and cyan sticks, respectively. (C) Electrostatic surface representation of the CupB6_dscB1_ structure with the two surface-exposed PPII helices in CupB6 shown as blue ribbons. Inset: a zoomed view of the major PPPPPIP segment with residues numbers indicated. (D) Top: Zoomed view of a single strand of helical collagen (magenta; from pdb: 3AH9[33]) manually docked into the groove between the two CupB6 PPII helices (blue) to mimic triple helical collagen. Bottom: Zoomed view of a surface representation of the CupB6_dscB1_ structure in the same orientation as in (C) with solvent exposed hydrophobic residues highlighted in orange. Residues delineating the hydrophobic pocket adjacent to the PPPPPIP helix are indicated.

Each molecule consists of two domains comprising a putative tip adhesin (residues; 1–202) and the pilin domain (202–343) (Fig. 2). The two domains are separated by a short linker. The pilin domain conforms to the classical Ig-like fold of other chaperone-usher pilin domains with a C-terminal β strand (strand G), provided by the NTE of CupB1 located at the C-terminus. Five alternating residues comprising glycine or large hydrophobic residues from the self-complementing G strand (G351, V353, F355, G357 and I359) are positioned to interact with the five pockets of the subunit groove (Fig. 1C) [4]. A search of the protein data back using the Dali server [17] reveals that the close structural homologues to CupB6 are the pilin subunits for the type I fimbriae (Fig. 3A); FimA and FimF structures superpose with an RMSDs of 2.4 Å and 2.6 Å over 146 equivalent residues of the polypeptide backbone [18], [19], [20]. Another notable similarity is identified with the equivalent pilin domain from the tip subunit LpfD from the long polar fimbriae protein of adherent-invasive *Escherichia coli* [21].

The adhesin domain of CupB6 is 205 amino acid residues in length and is significantly larger than typical adhesion domains of the chaperone-usher family, which are usually between 150 and 180 residues. The most similar structure determined to-date is that for the F17 family of fimbriae from enterotoxigenic *E. coli* (Fig. 3B), which play a role in colonization of the digiform brush border microvilli of intestinal epithelia [22], [23]. The domains superpose with an RMSD of 3.2 Å over 145 equivalent backbone residues. Flexible F17 fimbriae recognize distinct carbohydrate moieties and possess a different morphology to the rigid, rod-shaped type-1 and P pili from uropathogenic *E. coli* strains. Structures of the bound form of F17b-G lectin domain reveal a binding site for terminal *N*-acetyl d-glucosamine (GlcNAc) residues. Within the F17b-G binding site, a highly conserved tryptophan side chain (W109) provides an important stacking platform for the GlcNAc moiety and a neighboring residue of bound disaccharides (Fig. 3B). No aromatic residues are present in the CupB6 structure at similar positions and key hydrogen bonding side chains are also absent (e.g. D88). While the presence of the receptor-binding pocket at the apical tip of the adhesin domain in F17b-G is similar to that for FimH and PapG, their precise locations are distinct [3], [24]. The lack of a convincing binding pocket at the tip of the CupB6 adhesin domain would suggest it recognizes its receptor using an alternative surface. Several chaperone-usher fimbriae harbor binding sites for protein ligands, however these tend to be more diverse in terms of location and interacting residues. Some are polyadhesive and utilize surfaces of the main pilin subunit, such as in Aggregative Adherence Fimbriae from Enteroaggregative *E. coli* [3], [25], which binds the extracellular matrix protein fibronectin, and the *E. coli* Dr. family of afimbrial adhesins [26], [27], which recognizes both the decay-accelerating factor and CEA-related cell adhesion molecules using different surfaces. Others possess overlapping binding sites for both host carbohydrate and protein partners [24]. For example, FimH of type 1 pili has been shown to interact with Type IV collagen via a location distinct from that used to recognize mannosylated glycoproteins [28], [29]. One unique feature in the CupB6 structure is a surface-exposed polyproline helix (^199^PPPPPIP^205^) at the C-terminus of the adhesion domain (Figs. 2A, B; 3C). Adjacent to this is a shorter sequence (^17^LPWR^19^) that also adopts PPII helical secondary structure despite containing only one central proline. Despite a low abundance in folded domains, polyproline II (PPII) helices are often associated with roles in protein-protein interfaces. Adjacent to the PPII helix in CupB6 is a shallow pocket delineated by hydrophobic residues V21, L25, F36 and I204 (Fig. 3D), which further suggests a role in receptor binding. A prominent example of PPII motifs mediating host-bacteria interactions is the interaction between ActA of the food-borne pathogen *Listeria monocytogenes* and EVH1 domain of Mena, which regulates mammalian cytoskeleton [30]. Another striking example of a PPII helix in a bacterial adhesin is the *Streptococcus mutans* antigen I/II (AgI/II), a cell surface-localized adhesin that interacts with the salivary pellicle. An extended α-helix makes a high affinity interaction with a PPII helix to form an intertwined stalk-like structure that projects the adhesive elements away from the bacterial surface. *Pseudomonas aeruginosa* has long been known to degrade and bind to damaged basement membrane, and this includes an identified interaction with type IV and I collagen [31], furthermore a role for collagen glycosylation has also been implicated [32]. It is conceivable that CupB6 plays a role in mediating these interactions. The proximity and the surface-exposed PPII helices suggests that CupB6 could form triple-helix contacts with displaced polyproline strands in collagen regions. A single strand of collagen can be manually docked between the two CupB6 PPII helices using the arrangement present in the crystal structure of model triple helical collagen peptide as a template [33]. This places the free strand of the collagen receptor adjacent to the hydrophobic pocket delineated by V21, L25, F38 and I204 (Fig. 3D), which could accommodate a carbohydrate moiety. Further work is required to unveil the finer structural details and mechanism of the collagen binding capabilities of *Pseudomonas*. Our new structure of CupB6 provide a foundation for the design of insightful mutagenesis and functional experiments. The notion that CupB6 interacts with polyproline stretches of damaged collagen may provide clues for the design of new anti-adhesive therapeutics, perhaps using on peptidomimetic approaches.

## Transparency Document

Transparency document. Image 1

## Figures and Tables

**Table 1:** Crystallographic data and refinement statistics for CupB6_dsc_ protein.

Crystal parameters	Native	Se-peak	Se-inflection	Se-remote
Space group	*C2*	*C2*	*C2*	*C2*
Cell dimensions (Å)	a = 358.8, b = 88.9, c = 173.0 β = 113	a = 357.9, b = 89.4, c = 173.3 β = 113	a = 358.3, b = 89.5, c = 173.5 β = 113	a = 358.7, b = 89.5, c = 173.7 β = 113
Molecules per asymmetric unit	8	8	8	8

Date collection				
Beamline	DLS I02	DLS I02	DLS I02	DLS I02
Detector	Pilatus 6M-F	Pilatus 6M-F	Pilatus 6M-F	Pilatus 6M-F
Wavelength (Å)	0.92002	0.97903	0.97957	0.96757
Resolution (Å)	97.29–2.77 (2.84–2.77)	79.78–3.52 (3.72–3.52)	79.42–3.61 (3.80–3.61)	79.48–3.71 (3.91–3.71)
Unique observations	109,297 (8119)	58,155 (5247)	56,780 (7062)	51,573 (6059)
R_meas_	0.116 (1.011)	0.190 (0.755)	0.194 (0.763)	0.204 (0.754)
<* I* >/σ*I*	8.9 (1.4)	13.1 (2.4)	31.1 (2.6)	13.1 (2.7)
Completeness (%)	96.6 (96.9)	92.6 (57.7)	96.6 (83.5)	95.2 (77.7)
CC(1/2) [34]	0.970 (0.578)	0.994 (0.822)	0.994 (0.748)	0.992 (0.748)
Redundancy	3.4 (3.3)	5.0 (2.3)	4.8 (2.2)	4.9 (2.5)
Wilson B value (Å^2^)	57.4	41.1	43.9	44.1

Refinement				
R_work_/R_free_ (%)	21.7/24.7	–	–	–
Protein residues in asymmetric unit	2837	–	–	–
Water molecules in asymmetric unit	300			
Average B value (Å^2^)	56.7			

Rmsd stereochemistry				
Bond length (Å)	0.013	–	–	–
Bond angles (°)	1.789	–	–	–

Ramachandran analysis				
Residues in preferred regions	98.0%	–	–	–
Residues in allowed regions	2.0%	–	–	–
